# Modeling the Distribution of Migratory Bird Stopovers to Inform Landscape-Scale Siting of Wind Development

**DOI:** 10.1371/journal.pone.0075363

**Published:** 2013-10-02

**Authors:** Amy Pocewicz, Wendy A. Estes-Zumpf, Mark D. Andersen, Holly E. Copeland, Douglas A. Keinath, Hannah R. Griscom

**Affiliations:** 1 The Nature Conservancy, Wyoming Chapter, Lander, Wyoming, United States of America; 2 Wyoming Natural Diversity Database, University of Wyoming, Laramie, Wyoming, United States of America; Liverpool John Moores University, United Kingdom

## Abstract

Conservation of migratory birds requires understanding the distribution of and potential threats to their migratory habitats. However, although migratory birds are protected under international treaties, few maps have been available to represent migration at a landscape scale useful to target conservation efforts or inform the siting of wind energy developments that may affect migratory birds. To fill this gap, we developed models that predict where four groups of birds concentrate or stopover during their migration through the state of Wyoming, USA: raptors, wetland, riparian and sparse grassland birds. The models were based on existing literature and expert knowledge concerning bird migration behavior and ecology and validated using expert ratings and known occurrences. There was significant agreement between migratory occurrence data and migration models for all groups except raptors, and all models ranked well with experts. We measured the overlap between the migration concentration models and a predictive model of wind energy development to assess the potential exposure of migratory birds to wind development and illustrate the utility of migratory concentration models for landscape-scale planning. Wind development potential is high across 15% of Wyoming, and 73% of this high potential area intersects important migration concentration areas. From 5.2% to 18.8% of each group’s important migration areas was represented within this high wind potential area, with the highest exposures for sparse grassland birds and the lowest for riparian birds. Our approach could be replicated elsewhere to fill critical data gaps and better inform conservation priorities and landscape-scale planning for migratory birds.

## Introduction

Conservation of migratory birds requires an understanding of habitat, behavior and threats faced by birds during breeding, wintering, and migration. Migration is the most poorly understood of these annual activities, and of particular importance is understanding the distribution of stopovers and pathways used by migrating birds [1]. Recent technological advances, including telemetry devices, radar, stable isotope analysis, and genetic markers, permit the tracking of birds during migration [2]. Geographic Information System (GIS) modeling is also being used increasingly across large regions to evaluate conservation strategies and assess risks to migrating birds [3], [4].

One risk to migrating birds is wind energy development, which is expected to increase substantially in the United States in the coming decades due to evolving policies aimed at increasing renewable energy production [5]–[7]. Wind development can negatively impact birds through direct mortality from turbine collisions, avoidance behavior, and indirect effects of habitat fragmentation [8]–[12]. The U.S. Fish and Wildlife Service, Partners in Flight, The Wildlife Society, and the American Bird Conservancy, among others, have raised concerns about the long-term impacts of wind energy on bird populations [9], [13]. Mortality related to wind turbines could have especially great effects on declining species and long-lived species with low fecundity, such as raptors [14].

Wind development impacts to migratory birds may be reduced if facilities avoid major migration stopovers and flyways or if turbine operations are reduced in these areas during peak migration [13], [15]. However, the lack of information on the distribution of migratory concentration areas, and their overlap with wind energy resources, impedes conservation and proactive development planning [16]. Several studies have examined bird migration patterns and modeled stopovers and pathways in the eastern U.S. [3], [4], but much less is known about migration patterns in the western U.S. [17], especially in the Rocky Mountains. Limited regional information exists as incidental sightings [18], migration counts [19], [20], local or species specific research reports, e.g. [21]–[23], and expert knowledge, but has not been synthesized.

We developed a deductive modeling approach based on a synthesis of literature and expert knowledge concerning bird migration, and represented through GIS datasets, to map migratory concentration areas across the state of Wyoming. We produced deductive models due to concerns regarding the quality and quantity of available occurrence data needed to generate reliable inductive models. Deductive models, often referred to as habitat suitability models, are based on knowledge from literature or experts that is represented directly via environmental variables, while inductive models relate environmental variables to species occurrence locations using statistical algorithms [24]. Researchers have begun generating nationwide models depicting species’ distributions throughout the year based on inductive modeling of occurrences [25]; these efforts contribute significantly to our understanding of migration timing at broad scales. However, these efforts are limited by a lack of occurrence data from migration seasons for sparsely populated areas like Wyoming, and by the inclusion of only a few general predictors of distribution. We were able to identify, create, and tune model parameter layers (e.g., topographic leading lines) that represent important drivers of local migratory concentration. It will likely be many years before there is sufficient occurrence data to model migration concentration across Wyoming using inductive methods, and there is an urgent need for these models now.

The goals of our research were to 1) create and test spatially-explicit models representing migratory concentration areas for four functional bird groups and 2) assess the potential exposure of bird migration concentration areas to future wind energy development, to illustrate the utility of migration concentration models for landscape-scale planning. Wyoming has abundant wildlife resources, relatively intact ecosystems, and also some of the nation’s best wind energy resources. Wyoming currently has nearly 1000 wind turbines, and an additional 5000 turbines could be installed during the next 20 years [26]. Wind development has the potential to impact bird populations within the state and beyond its borders, if development occurs without regard for migrating birds. The migratory concentration maps presented here provide preliminary data to companies and land management agencies planning for wind development in Wyoming, and our methods could be replicated in other places where maps of migration hotspots are lacking.

## Methods

### Study Area

**Figure 1:**
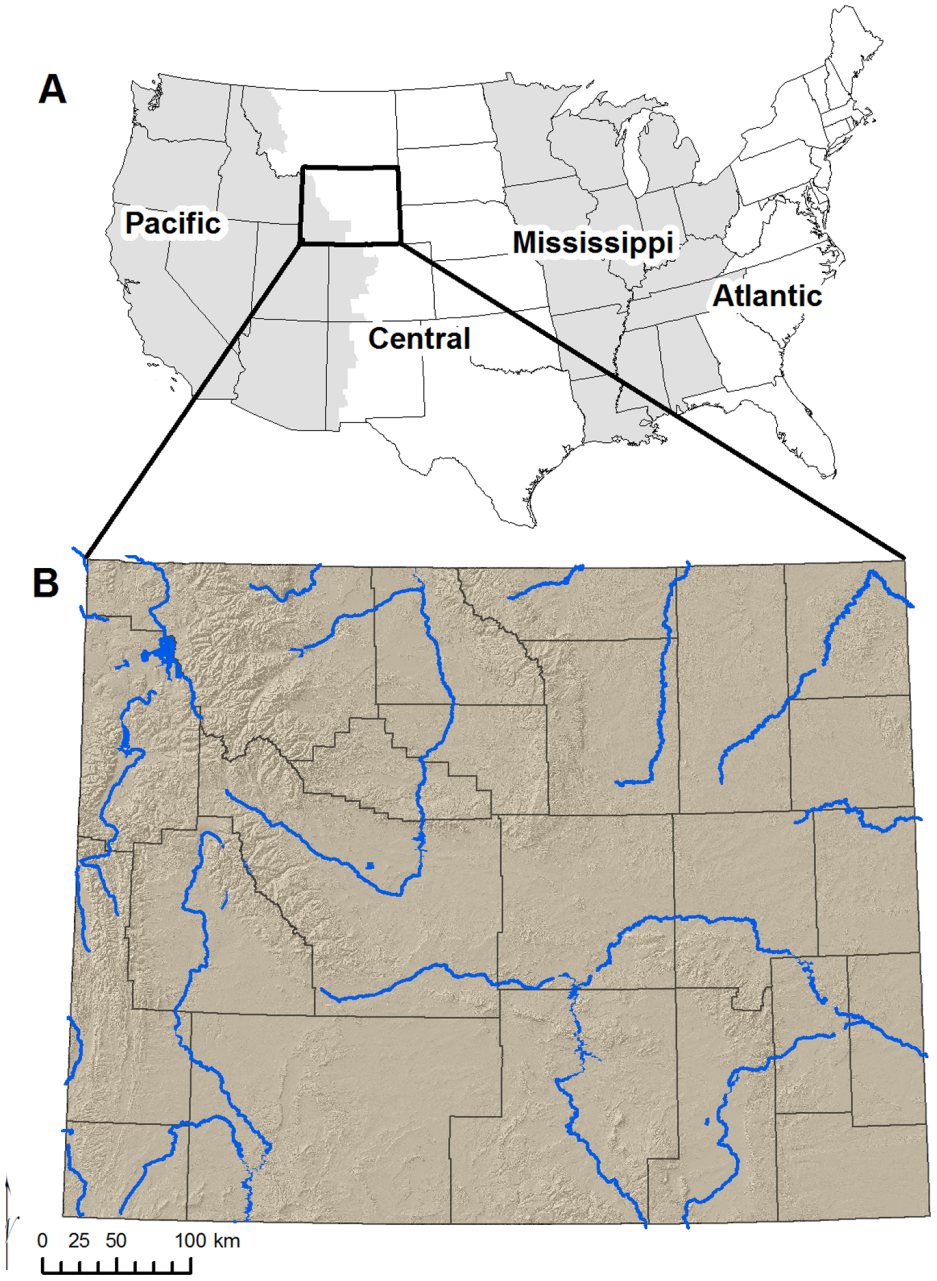
Features influencing bird migration in Wyoming. (A) Wyoming lies on the western edge of the Central Flyway and eastern edge of the Pacific flyway. Flyways are identified in varying shades of gray. (B) Key features influencing bird migration include topography (shown in brown shading) and major rivers (shown in blue). County boundaries are displayed for reference.

Our study area encompasses the state of Wyoming, which lies on the boundary between the Central and Pacific Flyways (Figure 1). Wyoming’s several large mountain ranges are dominated by conifer forests and are the source of several major rivers. Sagebrush and other shrublands dominate the inter-mountain basins, and grasslands are found in the lowest elevations of eastern Wyoming.

Wyoming is the least populated state in the United States. Lands are primarily used for livestock grazing in the western two thirds of the state and for both crop production and grazing in the eastern third. Despite Wyoming’s low human population, much of the state is experiencing energy development [27]. In addition to extraction of fossil fuels, including coal, oil, and natural gas, Wyoming has received considerable interest in its wind energy potential recently due to wind resources that rank it 8^th^ out of the 50 U.S. states [28].

### Modeling Bird Migratory Concentration

We created models representing where four functional groups of birds concentrate in Wyoming during their migration. We focused primarily on groups of birds species that concentrate at stopovers during migration to stage, forage or rest [29], because migrants that are concentrated in large densities are at greater risk for collisions with wind turbines [15], [30]. We used functional groups having similar migration behaviors, because insufficient migratory behavior information and occurrence data are available for many individual species. The four functional groups were wetland birds, riparian birds, raptors and sparse grassland birds, and species represented by each group are listed in Tables 1 and 2. Sparse grassland birds are those species that use sparsely-vegetated grasslands. All groups are comprised of species that concentrate during migration, except sparse grassland birds, which were included because many of these species are declining. We modeled spring migration patterns for wetland and riparian birds and fall migration patterns for raptors, because migration is most concentrated during these seasons for each group. We did not model both spring and fall migration for these groups, because we were most interested in when birds are most concentrated. For sparse grassland birds, we modeled spring migration because preliminary analysis indicated that our model was more indicative of spring than fall migrant distribution. We initially considered forest and shrubland birds but did not include them because they concentrate less during migration and may be partly represented by the riparian group, because they often follow riparian corridors during migration [17], [21], [22], [31].

**Table 1:** Wetland and riparian bird species represented by the migration concentration models, migration time periods for each species, and the numbers of observations used from existing occurrence datasets for model validation.

Species name	Migration time period (month-day)	Number of validation observations
**Wetland birds** a		54
Common Loon *(Gavia immer)*	04-01 to 05-22	3
Clark’s Grebe *(Aechmophorus clarkii)*	04-01 to 05-22	3
Double-crested Cormorant *(Phalacrocorax auritus)*	03-15 to 05-22	2
Great Blue Heron *(Ardea herodias)*	03-08 to 05-08	3
Snowy Egret *(Egretta thula)*	04-22 to 05-22	3
Black-crowned Night-Heron *(Nycticorax nycticorax)*	03-22 to 05-22	3
White-faced Ibis *(Plegadis chihi)*	03-15 to 05-15	3
Northern Pintail *(Anas acuta)*	02-15 to 03-31	3
Lesser Scaup *(Aythya affinis)*	03-01 to 06-15	3
Canvasback *(Aythya valisineria)*	03-01 to 05-08	3
Redhead *(Aythya americana)*	02-22 to 05-15	3
Barrow’s Goldeneye *(Bucephala islandica)*	04-22 to 06-01	3
Virginia Rail *(Rallus limicola)*	04-01 to 05-15	2
Sandhill Crane *(Gras canadensis)*	03-01 to 05-22	3
American Avocet *(Recurvirostra americana)*	03-15 to 05-15	3
Franklin’s Gull *(Leucophaeus pipixcan)*	04-01 to 05-22	2
Caspian Tern *(Hydroprogne caspia)*	04-01 to 05-15	3
Black Tern *(Chlidonias niger)*	05-01 to 05-31	3
Forster’s Tern *(Sterna forsteri)*	04-15 to 05-15	3
**Riparian birds**		51
Black-billed Cuckoo *(Coccyzus erythropthalmus)*	05-15 to 06-01	0
Yellow-billed Cuckoo *(Coccyzus americanus)*	05-01 to 05-31	0
Willow Flycatcher *(Empidonax traillii)*	05-01 to 05-31	4
Yellow Warbler *(Setophaga petechia)*	05-01 to 05-31	10
MacGillivray’s Warbler *(Geothlypis tolmiei)*	04-15 to 05-31	0
Yellow-breasted Chat *(Icteria virens)*	05-01 to 05-31	5
Blue Grosbeak *(Passerina caerulea)*	05-15 to 07-07	10
Song Sparrow *(Melospiza melodia)*	03-15 to 05-15	10
Lincoln’s Sparrow *(Melospiza lincolnii)*	04-15 to 05-30	2
Orchard Oriole *(Icterus spurius)*	05-01 to 05-31	0
Bullock’s Oriole *(Icterus bullockii)*	04-22 to 06-01	10

a The wetland bird model represents a large number of species. To maintain consistent datasets among the groups for the validation, we only included in the validation species of conservation concern that also had available occurrence data. The additional species represented by the model are: American Bittern *(Botaurus lentiginosus),* Eared Grebe (*Podiceps nigricollis*), Pied-billed Grebe (*Podilymbus podiceps*), Western Grebe (*Aechmophorus occidentalis*), American White Pelican (*Pelecanus erythrorhynchos*), Canada Goose (*Branta canadensis*), Mallard (*Anas platyrhynchos*), Gadwall (*Anas strepera*), American Wigeon (*Anas americana*), Northern Shoveler (*Anas clypeata*), Blue-winged Teal (*Anas discors*), Cinnamon Teal (*Anas cyanoptera*), Green-winged Teal (*Anas crecca*), Ring-necked Duck (*Aythya collaris*), Bufflehead (*Bucephala albeola*), Common Merganser (*Mergus merganser*), Ruddy Duck (*Oxyura jamaicensis*), Sora (*Porzana carolina*), American Coot (*Fulica americana*), Black-necked Stilt (*Himantopus mexicanus*), Spotted Sandpiper (*Actitis macularia*), Wilson’s Phalarope (*Phalaropus tricolor*), Ring-billed Gull (*Larus delawarensis*), and California Gull (*Larus californicus*).

**Table 2:** Raptor and sparse grassland bird species represented by the migration concentration models, migration time periods for each species, and the numbers of observations used from existing occurrence datasets for model validation.

Species name	Migration time period (month-day)	Number of validation observations
**Raptors**		52
Cooper’s Hawk *(Accipiter cooperii)*	08-15 to 11-15	4
Sharp-shinned Hawk *(Accipiter striatus)*	10-22 to 12-22	7
Northern Goshawk (*Accipiter gentilis)*	11-15 to 12-15	1
Red-tailed Hawk *(Buteo jamaicensis)*	08-01 to 11-01	10
Golden Eagle *(Aquila chrysaetos)*	09-15 to 12-31	10
American Kestrel (*Falco sparverius)*	08-15 to 09-21	10
Merlin *(Falco columbarius)*	09-01 to 10-22	10
**Sparse grassland birds**		51
Swainson’s Hawk *(Buteo swainsoni)*	04-01 to 05-15	7
Ferruginous Hawk *(Buteo regalis)*	03-01 to 03-31	7
Mountain Plover *(Charadrius montanus)*	03-01 to 04-15	7
Burrowing Owl *(Athene cunicularia)*	03-01 to 04-30	7
Horned Lark *(Eremophila alpestris)*	03-15 to 04-31	7
Lark Bunting *(Calamospiza melanocorys)*	04-01 to 05-31	4
Grasshopper Sparrow *(Ammodramus savannarum)*	05-01 to 05-31	7
McCown’s Longspur *(Rhynchophanes mccownii)*	03-01 to 04-31	5
Chestnut-collared Longspur *(Calcarius ornatus)*	03-01 to 04-15	0

Our models were based on the stopover ecology and migration behavior of migrants derived from peer-reviewed and gray literature [8], [17], [21]–[23,29–76]. For each group, this gathered information was synthesized to identify and rank key factors important for migration and additional variables that modify factor importance in certain locations. The general form of the models is shown in Equation 1, where MIS_c_ is the migratory importance score for raster cell c, f_ci_ are factors important for migration (e.g., wetlands), m_i_ are modifiers that changed the importance of factors under specific conditions (e.g., an elevation modifier might decrease the importance of high-elevation wetlands relative to low-elevation wetlands), w_i_ are weights that allowed some modified factors to be assigned greater relative importance than others, and n is the number of factors considered in the model.
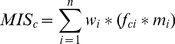
(1)


The conceptual models were represented spatially using raster-based GIS data, at 90-m resolution, through commonly-applied methods for integrating multiple factors into suitability maps [77]. Raster cells in datasets representing factors (e.g., streams) were scaled from 0 to 1, where 0 = no importance for migration, 0.25 = low importance, 0.5 = medium importance, and 1 = high importance. Raster cells in datasets representing modifiers of factors (e.g., orientation of streams) were assigned values ranging from 0 to 2, where values of 0 reduced the importance of associated factors to zero, values of 1 left associated factor values unchanged, and values of 2 doubled the importance of associated factors in those locations. After multiplication by one or more modifiers, the value of an individual factor was normalized to range from 0 to 1. This normalization assured that each factor had the same relative importance in the model. However, for factors identified as being of greater importance than other factors, we multiplied that factor by a weight greater than 1 [77]. Finally, the individual normalized weighted or unweighted factors were added to cumulatively represent factors important to migration concentration. The final MIS values were normalized to range from 0 to 1.

More than fifty Wyoming bird experts, who represented state and federal agencies, non-profit groups, and the University of Wyoming, were identified and invited to provide input on a an earlier version of the models presented in this paper. We received feedback and suggestions from more than 25 of these experts through in-person and phone meetings and written comments, and we made modifications to the models based on this input.

#### Wetland bird migration concentration

The wetland bird group includes waterfowl as well as birds that feed by wading in shallow water and mudflats along the shore of wetlands (hereafter “shorebirds”; Table 1). We identified that streams, wetland density, wetland size, and forage availability are important factors for wetland bird spring migration concentration, and that the importance of these factors varies with elevation, proximity to rivers, and location within or outside the Central Flyway. The model was implemented as: w_1_*(Streams)+w_2_*(Wetland density * *Elevation* * *Proximity to river* * *Flyway location*)+w_3_*(Wetland size * *Elevation* * *Proximity to river* * *Flyway location*)+w_4_*(Forage availability * *Proximity to river*)+w_5_*(Take-off/approach buffer), where modifiers are italicized and w_1_, w_3_, w_4_, w_5_ = 1 and w_2_ = 3. Value ranges of model factors and modifiers are described in Table 3.

**Table 3:** Descriptions of variables used in the model of wetland bird migration concentration.

Factor or modifier levels	Value and description	Data sources
**Streams** (Factor)		National Hydrology Dataset
Large rivers (i.e. North Platte, Green)	1: High importance	
Perennial & intermittent, order 3 & 4	0.5: Medium importance	
Perennial & intermittent, order 2	0.25: Low importance	
All other locations	0: No importance	
**Wetland density** (Factor)		National Wetlands Inventory;
At least 2 wetlands/km^2^ (per 5-km radius)	0.25–1: Importance increases linearly withdensity, where 0.25 = 2/km^2^ and 1 = 28/km^2^	Number of lacustrine or palustrine wetlands [96]
<2 wetlands/km^2^ (per 5-km radius)	0: No importance	
**Wetland size** (Factor)		National Wetlands Inventory; Freshwater emergent and forested/shrub wetlands and ponds and lakes
>15 ha	1: High importance	
5–15 ha	0.5: Medium importance	
<5 ha	0: No importance a	
**Forage availability** (Factor)		USDA National Agricultural Statistics Service CropScape (2010)
Grain crops Hay, pasture, non-grain crops	1: High importance 0.5: Medium importance	
All other locations	0: No importance	
**Take-off/Approach buffer** (Factor)		
1-km buffer (wetland size, streams)	Same value as stream or wetland	
Areas beyond 1-km buffers	0: No importance	
**Elevation** (Modifier)		National Elevation Dataset
<2438 m	1: Factor maintained	
2438–2743 m	0.5: Factor reduced	
>2743 m	0.1: Factor reduced	
**Proximity to river** (Modifier)		National Hydrology Dataset;
<5 km	2: Factor increased	Stream orders 3 and above
5–10 km	1.5: Factor increased	
>10 km	1: Factor maintained	
**Flyway location** (Modifier)		Central flyway represented by High Plains, Northwestern Great Plains, and Black Hills ecoregions [97]
Central flyway	1.25: Factor increased	
All other locations	1: Factor maintained	

a Wetlands <5 ha were not valued in the wetland size category; however, these small wetlands were included in the wetland density calculation.

Wetland birds generally migrate at night, stopping and feeding during the day. Wetland birds are among the species that attain the highest altitudes during migration, and are often capable of flying long distances (>3,200 km) non-stop unless forced by exhaustion or weather to land [32]. Wetland birds often use rivers to navigate during migration [32]–[34]. Rivers in the arid west may not only serve as navigation aids, but also a source of reliable stopover habitat in the form of reservoirs, off-channel wetlands, and agricultural fields [33], [35], [36]. Stopover habitat is typically similar to breeding habitat for a given species, though a wider range of resources may be used during migration [37]–[41]. Thus, stopover habitat for wetland birds includes marshes, wetlands, lakes, reservoirs, and other water bodies, e.g. [32], [33], [41]–[49]. In Wyoming, wetland birds concentrate in locations where wetlands are clustered in high densities. We weighted wetland density higher than other factors because its importance was emphasized by experts. This importance is also supported by the establishment of hundreds of national wildlife refuges for waterfowl encompassing important wetland clusters, and the use of these and similar wetland preserves by migrating waterfowl and shorebirds [32], [46], [50]. Larger lakes and wetlands can support large groups of migrating birds and provide safety from predators and are valuable even if not part of a wetland cluster. Heavy wing-loading in many wetland bird species can result in slow climbing rates [29], [32], placing them at risk of collisions with turbines during approach and take-off at stopover and foraging sites [30], [51]. We buffered wetlands and streams by 1 km to account for long approach/take-off distances needed by many wetland birds [8]. Agricultural lands can also provide food for migrating birds at stopover sites [52]. Many species of ducks, geese, and gulls forage in agricultural areas with grain crops [32], [53]–[56], and some wetland birds forage in irrigated pasture or hay meadows [47], [48].

The importance of wetlands and foraging areas varies with location. Wetlands and foraging areas closer to major streams are more likely to be used because of the tendency of wetland birds to travel along rivers. Wetland birds are unlikely to use high elevation wetlands during spring migration, because they may still be covered with snow or ice. We reduced the importance value for clusters of wetlands when they were located at high elevations that would likely be under snow cover during spring migration, using an elevation cutoff suggested by wetland bird experts. Eastern Wyoming overlaps the Central Flyway, a major migration route for waterfowl, and thus tends to have higher concentrations of ducks and geese than the western portion of the state.

#### Riparian bird migration concentration

The riparian bird group includes cuckoos and certain species of songbirds and flycatchers (Table 1). However, the model will over-predict migration habitat for birds restricted to mature cottonwood forests, such as the Yellow-billed Cuckoo (*Coccyzus americanus*). Our riparian model may also represent some forest or shrubland migrants, which often follow riparian corridors [17], [21], [22], [31]. We identified that streams and wetland density are important factors for riparian bird spring migration concentration, and that the importance of these factors varies with stream orientation, willow and cottonwood abundance, riparian structural diversity, elevation, and proximity to rivers. The model was implemented as: w_1_*(Streams * *Stream orientation* * *Cottonwood abundance* * *Willow abundance* * *Riparian structural diversity*)+w_2_*(Wetland density * *Elevation* * *Proximity to river*), where modifiers are italicized and w_1_ = 2 and w_2_ = 1. Value ranges of model factors and modifiers are described in Table 4.

**Table 4:** Descriptions of variables used in the model of riparian bird migration concentration.

Factor or modifier levels	Value and description	Data sources
**Streams** (Factor)		National Hydrology Dataset; Applied distance decay function (inverse distance squared) to provide some value to areas within 500 m of streams without adding a true buffer
Perennial streams (order 2–7)	1: High importance	
Intermittent streams (order 2–7)	0.25: Low importance	
Other streams (order 0–1)	0: No importance	
**Wetland density** (Factor)		National Wetlands Inventory;
At least 2 wetlands/km^2^ (per 5-km radius)	0.25–1: Importance increases linearly withdensity, where 0.25 = 2/km^2^ and 1 = 28/km^2^	Number of lacustrine or palustrine wetlands [96]
<2 wetlands/km^2^ (per 5-km radius)	0: No importance	
**Cottonwood abundance** (Modifier)		Cottonwood probability map [98] based on GAP Ecological Systems [99]
Cottonwood index	1–2, where 2 is most likely to havecottonwoods (factor increased)	
**Willow abundance** (Modifier)		Willow probability map [98] based on GAP Ecological Systems [99]
Willow index	1–2, where 2 is most likely to havewillows (factor increased)	
**Riparian structural diversity** (Modifier)		GAP Ecological Systems [99]
Forest riparian	2: Factor increased	
Riparian woodland or shrubland	1.5: Factor increased	
Shrub riparian	1.25: Factor increased	
Grass riparian	1: Factor maintained	
**Proximity to river** (Modifier)		National Hydrology Dataset; Stream orders 3 and above
<5 km	2: Factor increased	
5–10 km	1.5: Factor increased	
>10 km	1: Factor maintained	
**Elevation** (Modifier)		National Elevation Dataset
<2438 m	1: Factor maintained	
2438–2743 m	0.5: Factor reduced	
>2743 m	0.1 Factor reduced	
**Stream orientation** (Modifier)		Drew a minimum area rectangle around each stream. Calculated orientation of the long axis of each rectangle to determine stream orientation.
Northness index	1–2, where 2 = north/south (factor increased);1 = east/west (factor maintained)	

Riparian migrants concentrate along perennial streams where well-developed and structurally-diverse riparian trees and shrubs are present [17], [21], [22], [57], [58]. Riparian migrants are most likely to use larger, north-south oriented streams to guide migration [17], [58], and cottonwood and willow-dominated riparian areas are used more frequently than other vegetation types [17]. Isolated oases of riparian habitat are often found around large permanent wetlands and are important to riparian migrants, especially in arid landscapes like much of Wyoming [17], [58]; riparian birds will concentrate at permanent wetlands because of their riparian vegetation. Since riparian birds concentrate along large perennial streams, wetlands closer to these streams are more likely to be encountered and used as stopover habitat. Migrating birds will use all riparian areas in xeric landscapes, but lower elevation riparian corridors tend to be used by a greater number of species [22], [59]. We used stream order as a surrogate for an elevation cutoff for streams, to avoid excluding large streams that occur at high elevations. Some migrants may use different routes in spring and fall, with lower elevation riparian corridors used more heavily in spring, when higher elevation riparian and forested areas are still snow-covered with fewer food resources [17], [60], [61]. We reduced the importance of wetlands when they were located at high elevations that would likely be under snow cover during spring migration. The stream factor was given twice the weight of the wetland density factor in our model, to reflect the especially high importance of streams and riparian areas to this group of birds [17], [21], [58].

#### Raptor migration concentration

The raptor group includes diurnal birds of prey (Table 2). We identified that topographic features, updrafts, thermals, and streams are important factors for raptor fall migration concentration, and that the importance of these factors varies with topography and stream orientation and cottonwood abundance along streams. The model was implemented as: w_1_*(Topography * *Topography orientation*)+w_2_*(Updrafts)+w_3_*(Thermal formation)+w_4_*(Streams * *Stream orientation* * *Cottonwood abundance*), where modifiers are italicized and w_1_ through w_4_ = 1. Value ranges of model factors and modifiers are described in Table 5.

**Table 5:** Descriptions of variables used in the model of raptor migration concentration.

Factor or modifier levels	Value and description	Data sources
**Topography** (Factor)		Features were derived from a National Elevation Dataset using a topographic position index tool [100], using 20 and 50-cell circular windows
Tall ridges, foothills, hogbacks	1: High importance	
All other locations	0: No importance	
**Updrafts** (Factor)		Aspect was derived from National Elevation Dataset. Wind direction data was recorded at 10-m height over 12-km spatial resolution averaged for 2009 over the fall migration time period from August 27 to November 5 [19], using the Weather Research and Forecasting model [101].
Prevailing wind category = aspectcategory (i.e., 90° angle)	1: High importance	
Prevailing wind category within 1 category ofaspect (i.e., 45° angle)	0.75: Medium-high importance	
All other locations	0: No importance	
**Thermal formation** (Factor)		Bare ground cover index [98] was derived from LANDFIRE existing vegetation cover [102]. Cultivated croplands were added from USDA Agricultural Statistics Service CropScape (2010) data.
Bare ground index	0–1, where 1is mostlikely to have bare ground	
**Streams** (Factor)		National Hydrology Dataset; Applied distance decay function (inverse distance squared) to provide some value to areas within 500 m of streams without adding a true buffer
Large rivers (i.e., North Platte, Green)	1: High importance	
Perennial & intermittent, order 3 & 4	0.5: Medium importance	
All other locations	0: No importance	
**Topography or stream orientation** (Modifier)		Drew a minimum area rectangle around each feature. Calculated orientation of the long axis of each rectangle to determine orientation.
Northness index	1–2, where 2 = north/south(factor increased);1 = east/west (factor maintained)	
**Cottonwood abundance** (Modifier)		Cottonwood probability map [98]
Cottonwood index	1–2, where 2 is most likely to havecottonwoods (factor increased)	Based on GAP Ecological Systems [99]

Unlike many other migrants, most raptors do not maintain high altitudes during migration. Instead, they conserve energy by gaining lift from updrafts and thermals and gliding long distances, slowing losing altitude, to the next updraft or thermal [29], [62], [63]. Therefore, instead of concentrating at stopovers, raptors concentrate in areas that provide the best updrafts and thermals, especially during fall migration. Ridges and mountain ranges oriented perpendicular to prevailing winds produce the strongest updrafts. Although some ridges consistently provide strong updrafts, the location of updrafts can vary daily with local wind and weather conditions. As a result, when updrafts are not available, raptors will adjust their migration routes to take advantage of thermals, which form over surfaces that heat up the air faster (e.g. rock, sand, bare ground, pavement) [62], [63].

Prominent landscape features, including streams and topographic features such as tall ridges, provide leading lines that can guide raptor movements and concentrate migrants [62], [64]. Leading lines that are oriented in the general direction of migration (north/south in Wyoming) are of particular importance, as are stream leading lines that include perching locations such as cottonwood trees. Some raptor species avoid crossing inhospitable habitat, such as deserts and large water bodies, and divert travel around the edges of these features [62]. Both leading lines and diversion lines concentrate migrating raptors, but we focused on leading lines because Wyoming lacks substantial diversion lines.

Raptor species likely not well-represented by this model include the Prairie Falcon (*Falco mexicanus*) and Peregrine Falcon (*Falco peregrinus*); they migrate at much higher altitudes and have dispersed and unique migration patterns [65]–[67]. Bald Eagle (*Haliaeetus leucocephalus*) [23], Ferruginous Hawk (*Buteo regalis*) [68], and Swainson’s Hawk (*Buteo swainsoni*) [69] migration patterns also do not fit this raptor model due to specific habitat needs.

#### Sparse grassland bird migration concentration

The sparse grassland group includes species that use sparsely-vegetated grasslands or areas dominated by prairie dog colonies (Table 2). We identified that grassland land cover types and presence of prairie dogs are important factors for sparse grassland migration concentration, and that grasslands having more bare ground are preferred. The model was implemented as: w_1_*(Land cover * *Bare ground cover*)+w_2_*(Prairie dog occurrence likelihood), where modifiers are italicized and w_1_ and w_2_ = 1. Value ranges of model factors and modifiers are described in Table 6.

**Table 6:** Descriptions of variables used in the model of sparse grassland bird migration concentration.

Factor or modifier levels	Value and description	Data sources
**Land cover** (Factor)		
Short-grass or mixed-grass prairie	1: High importance	GAP Ecological Systems [99]
Medium or tall grasslands	0.5: Medium importance	
Arid shrublands	0.25: Low importance	
All other locations	0: No importance	
**Prairie dog occurrence likelihood** (Factor)		Merged predictive distribution models for white-tailed and black-tailed prairie dogs [98]
Prairie dog index	0–1, where 1 is most likely to have prairie dogs	
**Bare ground cover** (Modifier)		Bare ground cover index [98] was derived from LANDFIRE existing vegetation cover [102]
Bare ground index	1–2, where 2 is most likely to have bareground (factor increased)	

In Wyoming, the sparsely-vegetated grasslands used by these birds during migration are relatively widespread. Therefore, in Wyoming, this group of migrants tends to exhibit a more dispersed pattern during migration than other avian species. This model is based largely on broad habitat requirements, because specific information on migration behavior for this group was generally lacking. Sparse grassland birds prefer short-grass and mixed-grass prairie and/or low shrublands with a high bare-ground component [68], [70]–[73]. These species will often use heavily grazed, previously disturbed, tilled, and even somewhat degraded landscapes. Many sparse grassland birds, such as the Mountain Plover (*Charadrius montanus*), are often associated with prairie dog (*Cynomys* spp.) colonies because of the close-cropped grass and high bare-ground components they provide [73]–[76]. Prairie dog colonies also provide a diversity of small mammal and avian prey for raptors, such as the Ferruginous Hawk.

### Model Validation

The final predictive maps were validated using expert opinion and available observation data. Expert validation was completed through a web-based review of the final maps by statewide bird experts. We invited a larger group of experts to participate than for draft model feedback, but the majority of respondents had also been engaged in the first process. We asked experts to provide their assessment of each model on a 5-point Likert-type scale [78] that included rating selections of very poor (−1), poor (−0.5), okay (0), good (0.5), and very good (1) and to rate their level of expertise for each bird group on a 5-point Likert-type scale that included rating selections of very low (1), low (1.25), moderate (1.5), high (1.75), and expert (2). We multiplied the two rating values to weight each model rating by the reviewer’s level of expertise and averaged these expertise-adjusted scores for each model to obtain a final weighted-average score. Scores below zero indicated a poor model, scores of zero indicated an average model, and scores greater than 0 indicated a good model.

The observation-based validation used an occurrence dataset assembled from the Wyoming Natural Diversity Database (http://www.uwyo.edu/wyndd) and eBird [18] for each species represented by the models for the time period representing the majority of their typical spring or fall migration through Wyoming. From eBird, data were included only from exhaustive area, random, stationary and traveling counts. Migration time periods were extrapolated from species accounts in Birds of North America Online (http://bna.birds.cornell.edu/bna/species/), eBird [18], and Birds of Wyoming [79] (Tables 1 and 2). We removed occurrences of questionable quality or with a spatial accuracy of less than 400 m. Next, for each bird group, we removed points that were closer than 800 m to a point deemed to be of better quality, based on mapping precision, recentness, and certainty of identification. Filtered occurrence points were subsampled to balance contributions of individual species, to minimize bias of validation statistics towards particular species with more occurrences, while still providing a minimum of 50 points for validation that were well-distributed across Wyoming. For each bird group, validation points were selected at random from filtered occurrences, stratified by species (see Tables 1 and 2). Up to 10 points per species were selected, where available.

We applied the Boyce index to measure observed versus expected occurrence, using the selected validation data points and binned versions of the models. Bins were created so that each bin contained approximately the same number of validation points. The Boyce index is a Spearman rank correlation between the area-adjusted frequency of validation points falling within a bin and the associated bin’s rank [80]. The validation points were ranked based on their predicted concentration score, and we chose the midpoints of the scores above and below the bin breaks as binning thresholds for the raster models. The Boyce index varies between −1 (counter prediction) and 1 (positive prediction), with values close to zero indicating that the model does not differ from a random model. Data were partitioned into 10 bins for each group, based on the model value assigned to the validation points, with exception of riparian birds, which had 8 bins. The bins in the riparian model were more limited in number due to a large proportion of raster cells with a predicted concentration score of zero and thus a large number of points occurring in the first bin.

### Model Sensitivity and Uncertainty

We completed a sensitivity analysis of each of the four models to characterize the uncertainty associated with each model and describe how much the output of each model changed based on the contribution of each factor, modifier, or weight. We dropped each factor, modifier or weight one at a time from each model, and described the subsequent change in three ways. First, for each raster cell we calculated the percent difference between the partial model (missing one term) and the full model (all terms included), as the absolute value of the full model minus the partial model, divided by the full model. For each partial-model versus full-model combination, we calculated the mean and standard deviation of the percent difference across the study area. Second, we classified the full model raster and each partial model raster into 5-quantiles and, for each partial-model versus full-model combination, we tallied the number of raster cells having class agreement using the crosstab function in the R [81] raster package [82]. This resulted in an error matrix from which we calculated classification accuracy [83], the percentage of raster cells in each partial model that were classed in the same bin as the full model. Finally, to visualize potential spatial pattern in uncertainty, for each cell in our study area we calculated the mean of the percent difference values across all partial-model versus full-model combinations.

### Exposure of Migrants to Wind Energy Development

To assess the potential exposure of migratory birds to wind energy development, we measured the overlap between the maps of migration concentration and a predictive model of wind energy development potential. Our intent was to provide a coarse-scale analysis of where conflicts may exist with future wind development and to illustrate the utility of migration concentration models for landscape-scale planning. For these reasons, we used wind development potential rather limiting the analysis prescriptively to specific proposed wind farm projects. We created a predictive model of Wyoming wind energy development potential that incorporated wind resource potential, near-term development indicators and current development restrictions [84]. First, we fit a predictive model using maximum entropy methods [85], [86] and Maxent® software version 3.3.3e. Maxent uses presence-only data, which was appropriate for this dataset because we did not have true absence data representing where turbines could not feasibly be built. The model used existing wind turbines as the response variable [87]. Predictor variables were the average 50-m wind resource potential [88], percent slope, and topographic position (i.e., ridge, valley) [89], because these factors influence the quality of the wind resource or feasibility of turbine construction (see [84] for details). We used a randomly-selected 67% of wind farms (643 turbines, 32 farms) to train the model and 33% to test the model (319 turbines, 8 farms), including all turbines within individual wind farms as either training or test data to avoid spatial autocorrelation. The model performed well, with a test area under the receiver operating characteristic curve (ROC AUC) of 0.91. A ROC AUC value of 0.5 indicates model performance no better than chance and values above 0.5 indicate increasingly strong classification to an upper limit of 1 [90].

The Maxent® model represented the quality of wind resources but did not prioritize where development would most likely occur in the near term. Therefore, we adjusted the model results using short-term development indicators, including density of existing meteorological towers used to test wind speeds, distance to proposed transmission lines, proposed wind farm boundaries and land tenure [84]. Finally, we excluded locations where development was precluded due to legal or operational constraints, including protected lands (e.g. wilderness areas, conservation easements), airport runway space, urban areas, mountainous areas above 2743-m, and open water [84]. The adjusted model had a Boyce index of 0.89 (p = 0.001). A GIS version of the wind development model is available for download through the Wyoming Geographic Information Science Center (WYGISC).

We combined the wind potential dataset with each of the four migration model results to evaluate how much exposure migratory birds may have to future wind development. To understand spatial patterns in exposure, we first classified each of the five datasets into five quantile bins of potential for bird migration or wind development. This step was necessary to make the values comparable among the various models; while all models ranged from 0 to 1, the absolute values were scaled relative to each individual model. Values of 1, 0.75, 0.5, 0.25, and 0 were assigned to the quantile classes, where 1 corresponded to the quantile including the highest 20% of the data (i.e., very high). The wind potential and bird migration rasters with these new values were then multiplied, separately for each bird group. Where wind development potential was high (0.75) and migratory concentration was high (0.75), we assumed that exposure of birds to development would also be high (result = 0.5625) and that where wind development potential was low (0.25) and migratory concentration was low (0.25), exposure of birds would be low (result = 0.125). Therefore, we developed the following classes to reflect exposure level: very high (>0.75), high (0.56–0.75), moderate (0.26–0.559), low (0.1–0.259), or very low (<0.1). To spatially represent uncertainty in exposure, we determined exposure for each of the partial models and then calculated the standard deviation of the mean exposure across the full and partial models for each bird group.

Additionally, we focused on those areas with the highest likelihood for potential wind development – the top two quantile classes of high and very high – and summarized 1) the percentage of the top two migration quantiles for each bird group overlapping with these areas and 2) the percentage of the top two wind potential quantiles overlapping with the most important concentration areas for each bird group. We determined these percentages for the full models and also calculated the mean and 95% confidence interval across the full and partial models. Important migration concentration areas may not overlap spatially among the four bird groups, so we also combined the top two quantiles for each bird group into one raster representing cumulative migration concentration to generate the percentages described above.

## Results

**Figure 2:**
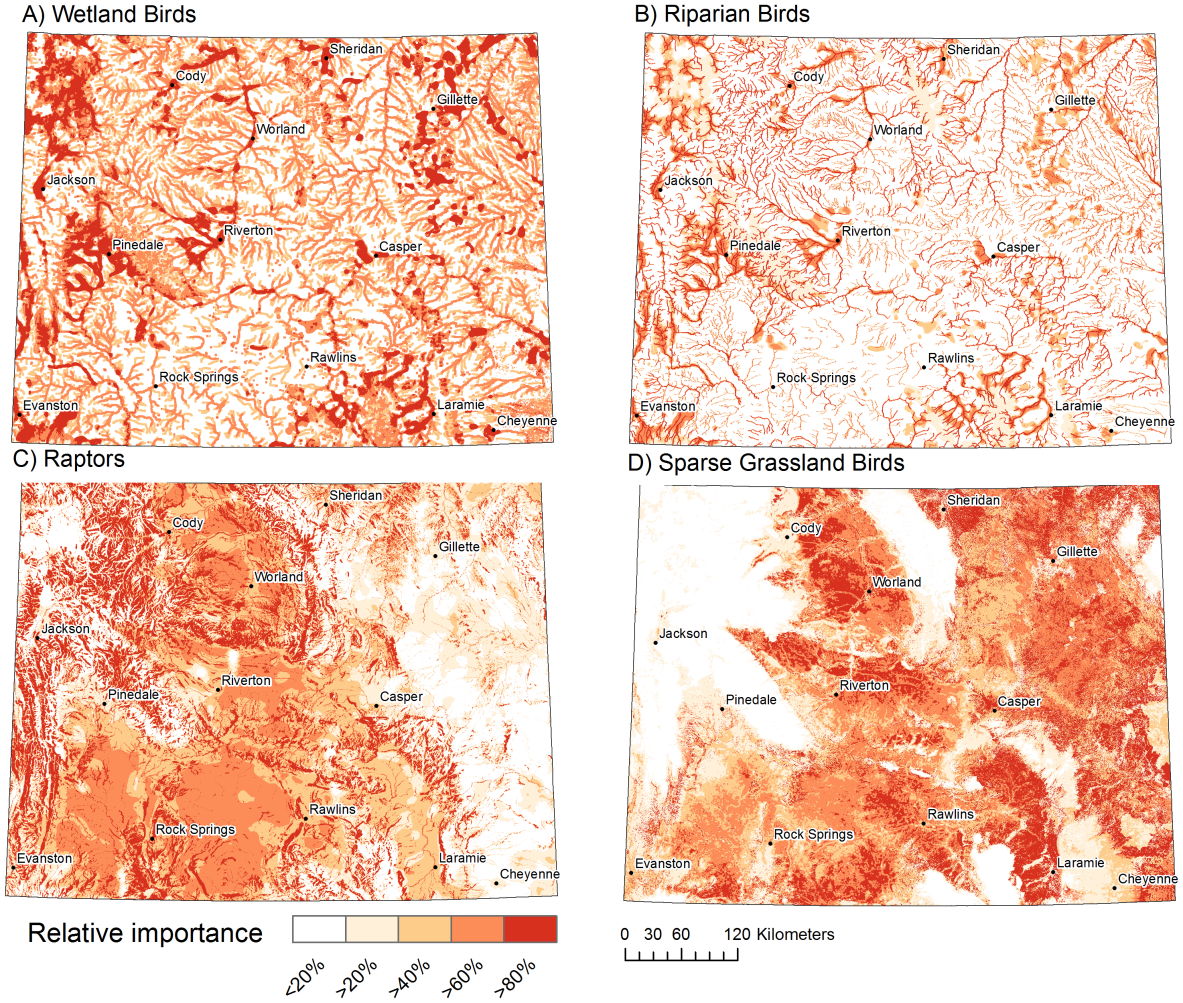
Modeled relative importance of migration concentration. Continuous modeled values were binned into five quantiles representing relative importance for migration concentration. Darker colors represent areas with greater importance, where >80% represents areas more important than those found across 80% of the state. The models represent (A) Raptor fall migration concentration (B) Wetland bird spring migration concentration (C) Riparian bird spring migration concentration and (D) Sparse grassland bird migration concentration.

The results for each model are presented as five quantiles in predictive maps, where the highest 20% of values are displayed as raster cells that are of greater importance for migration concentration than 80% of all cell locations (Figure 2). GIS versions of the migration models are available for download through WYGISC.

### Model Validation and Sensitivity

**Figure 3:**
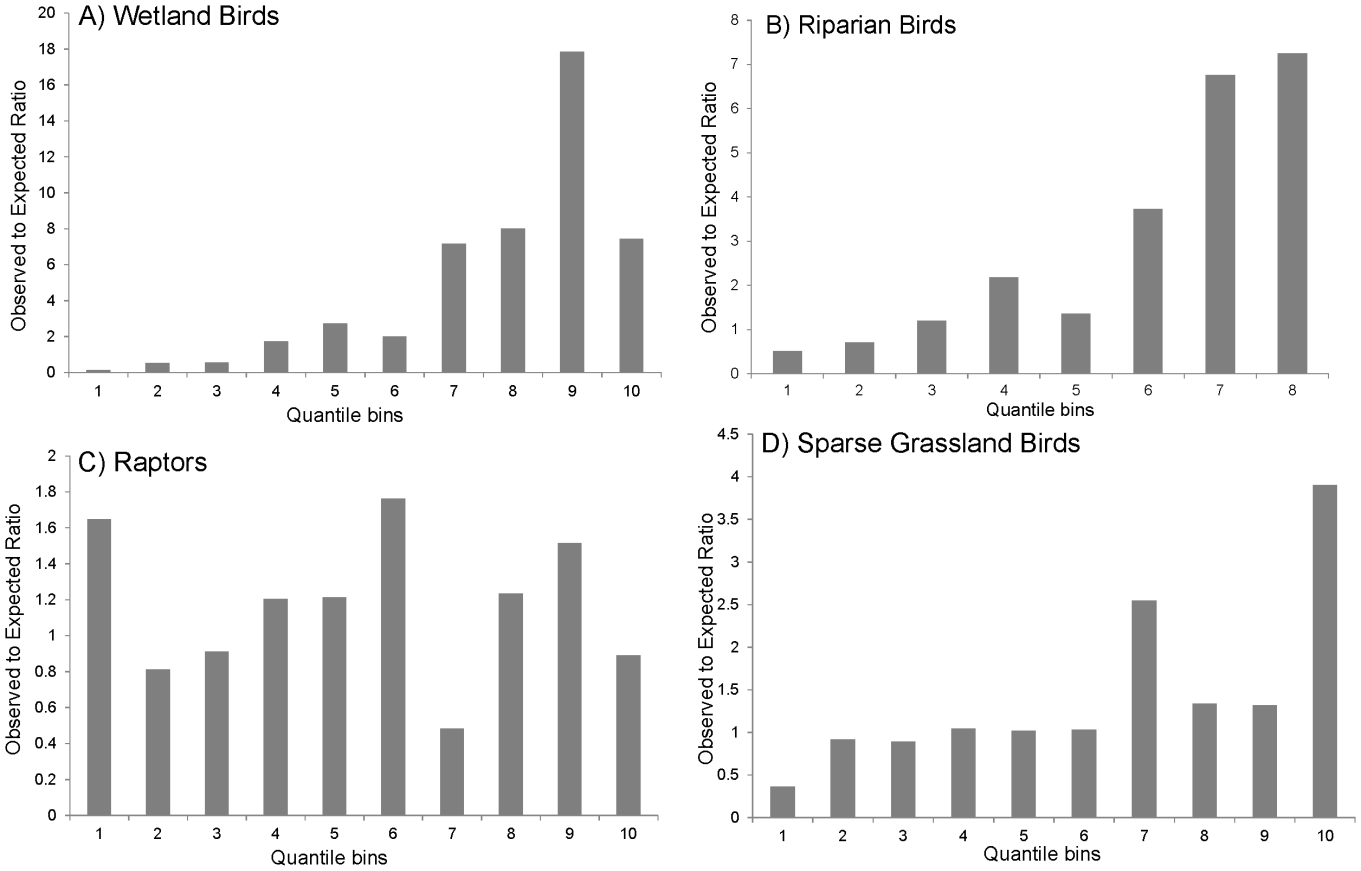
Model validation plots. The observed to expected ratio in each quantile bin used to calculate the Boyce index for validation of migration models for (A) wetland birds, (B) riparian birds, (C) raptors and (D) sparse grassland birds. Models with a perfect fit show a monotonic increase as bin numbers increase, which is best illustrated in panel A.

The expert validation survey was completed by 13 (28%) of the invited experts. An additional seven experts provided comments but did not rate the models. The overall model rating was “very good” for wetland (score = 0.88, n = 12) and riparian birds (score = 0.97, n = 11) and “good” for raptors (score = 0.45, n = 10) and sparse grassland birds (score = 0.69, n = 11). Qualitative comments were consistent with the aforementioned ratings, with experts providing the most favorable comments about the wetland and riparian bird models and raising more concerns related to the raptor and sparse grassland bird models. We found significant agreement between species occurrence data and the migration models for wetland (p = <0.0001, Boyce index = 0.952), riparian (p = 0.001, Boyce index = 0.976), and sparse grassland bird migration (p = <0.001, Boyce index = 0.903) (Figure 3). There was no agreement between occurrence data and the raptor migration model (p = 0.467, Boyce index = −0.030) (Figure 3).

**Figure 4:**
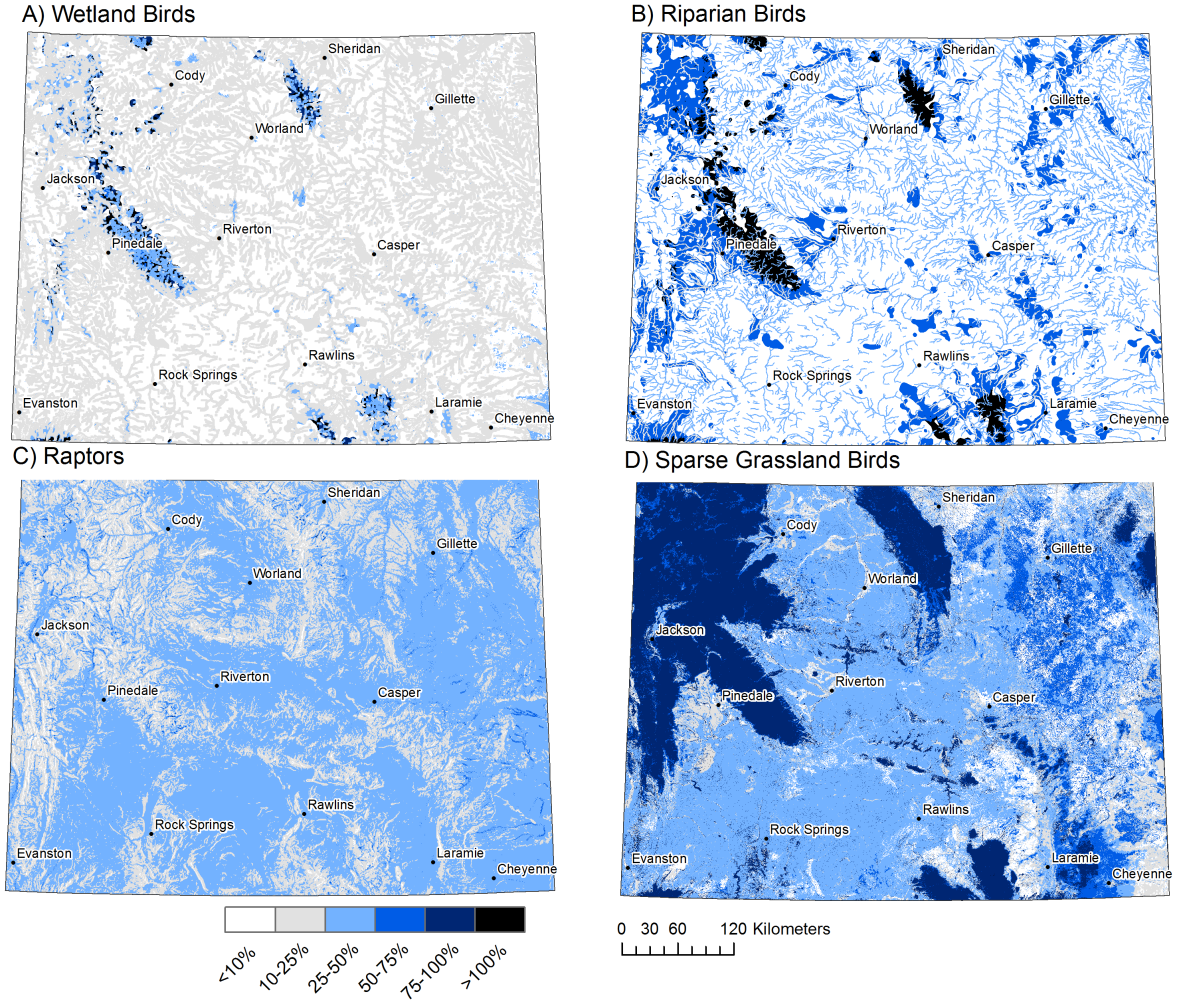
Model uncertainty. These values represent the average percent difference of the partial sensitivity models (with one variable dropped at a time) from the full models. Locations with higher values are locations where the various versions of the model had the greatest differences, for A) wetland birds, B) riparian birds, C) raptors, and D) sparse grassland birds.

Model were most sensitive to the removal of base factors, such as streams for riparian birds, wetland density for wetland birds, and land cover for grassland birds (Table 7). The raptor migration model was most sensitive to the updraft and thermal formation factors (Table 7). Overall, uncertainty was lowest for the wetland bird model and highest for the sparse grassland bird model (Figure 4a, d). Across models, uncertainty tended to be greatest at higher elevations (Figure 4a, b, d).

**Table 7:** Results of the model sensitivity analysis, where one term was dropped from the model at a time.

Factor, modifier or weight left out of the model	Mean (SD) of difference	Classification accuracy (%)
**Wetland bird model terms**		
Streams	11.6 (5.8)	98.6
Wetland density	47.5 (19.5)	67.1
Wetland density: elevation	44.0 (140.7)	87.1
Wetland density: river proximity	14.0 (5.7)	96.9
Wetland density: flyway location	12.0 (3.8)	97.8
Wetland size	11.4 (3.0)	99.3
Wetland size: elevation	18.6 (5.7)	99.7
Wetland size: river proximity	11.4 (3.0)	99.9
Wetland size: flyway location	18.2 (2.4)	99.8
Forage availability	4.6 (15.1)	97.9
Forage availability: river proximity	0.3 (3.9)	99.7
Take-off/approach buffer	70.5 (38.1)	47.2
Weight for wetland density	30.0 (10.6)	71.8
**Riparian bird model terms**		
Streams	121.8 (49.6)	73.5
Streams: orientation	53.4 (67.5)	81.4
Streams: cottonwood abundance	0.3 (0.9)	99.8
Streams: willow abundance	4.0 (2.4)	98.3
Streams: structural diversity	7.4 (5.6)	97.3
Wetland density	60.4 (100.0)	77.1
Wetland density: elevation	65.2 (164.7)	89.5
Wetland density: river proximity	60.4 (34.5)	98.0
Weight for streams	32.9 (14.4)	86.2
**Raptor model terms**		
Topography	33.4 (7.2)	79.2
Topography: orientation	10.5 (16.8)	75.6
Updrafts	34.4 (13.8)	41.0
Thermal formation	80.3 (26.1)	35.7
Streams	1.0 (3.9)	87.8
Streams: orientation	8.7 (19.0)	77.4
Streams: cottonwood abundance	14.5 (15.0)	76.5
**Sparse grassland bird model terms**		
Land cover	>1000 (>1000)	55.3
Land cover: bare ground	31.5 (64.2)	63.5
Prairie dog occurrence	56.8 (30.4)	53.1

For each raster cell we calculated the absolute percent difference of the new partial model relative to the full model and determined the mean and standard deviation (SD) of these differences across all cells for each pair. We classed each raster into 5-quantile bins and calculated classification accuracy, which represents the percentage of raster cells in the partial model that were classed in the same bin as in the full model.

### Exposure of Migrants to Wind Energy Development

**Figure 5:**
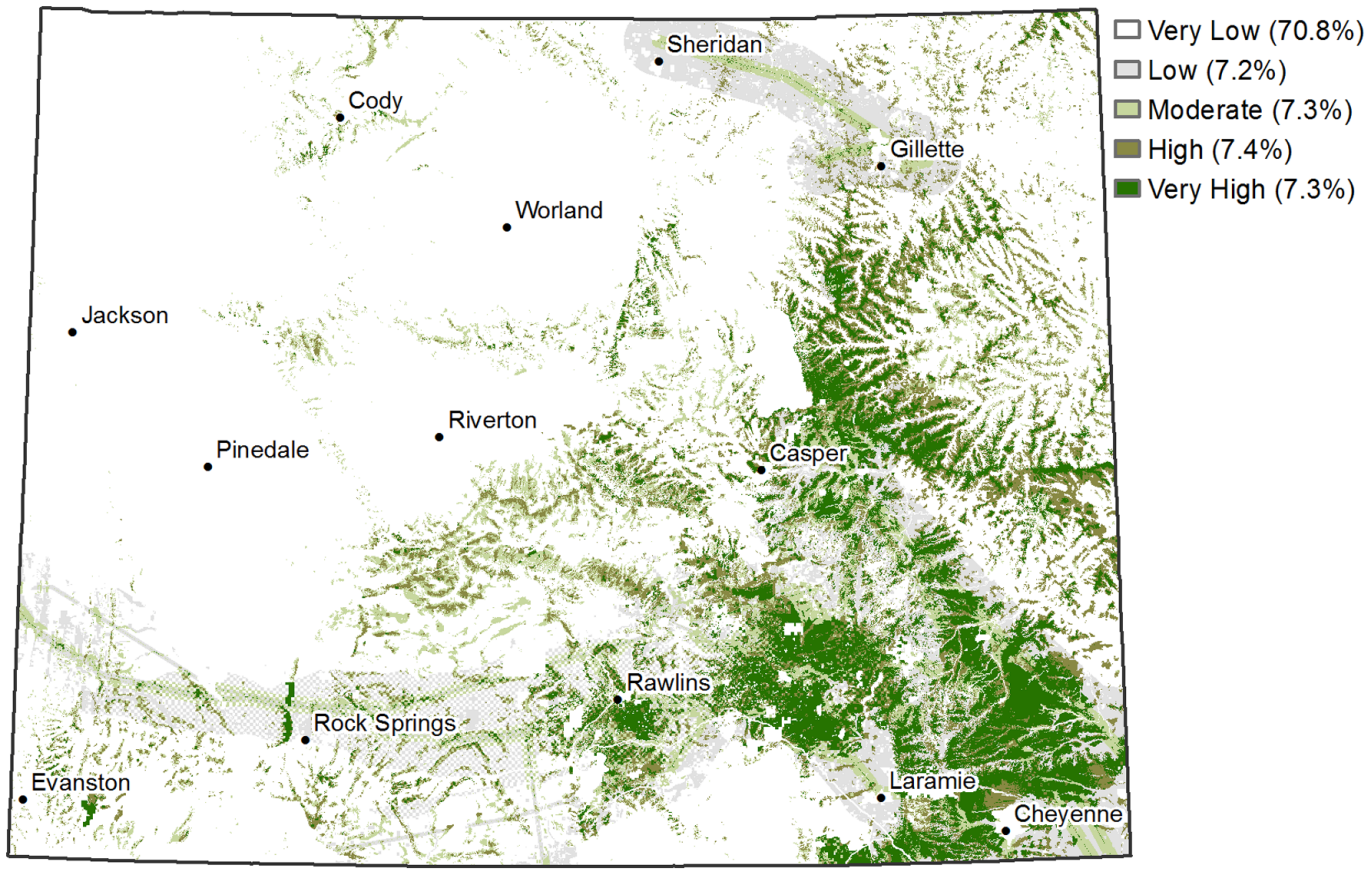
Predicted wind development potential across Wyoming. Continuous modeled values were binned into five quantiles representing relative development potential and are followed by the percentage of the state’s area included in that bin.

**Figure 6:**
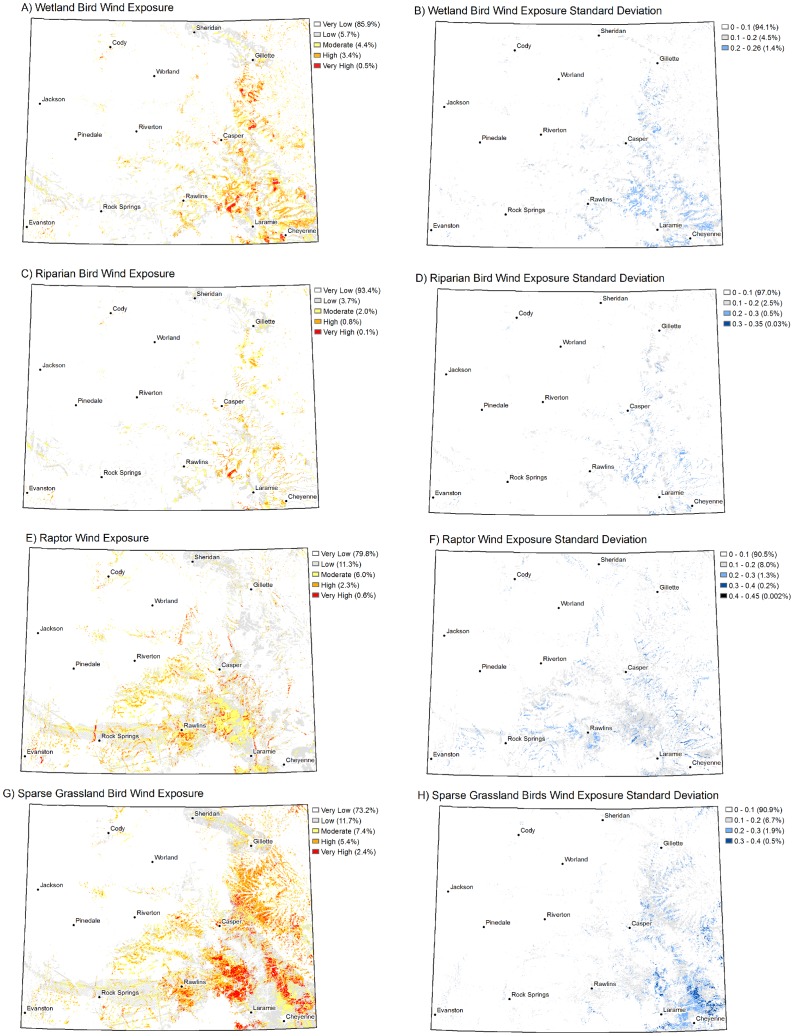
Exposure of bird migration concentration areas to potential wind development is shown for (A) wetland birds, (C) riparian birds, (E) raptors, and (G) sparse grassland birds. Exposure classes in each map are followed by the percent of the state occurring in that class. Uncertainty in exposure is represented by the standard deviation in exposure among the full and partial models for each bird group and is shown for (B) wetland birds, (D) riparian birds, (F) raptors, and (H) sparse grassland birds. Standard deviation is relative to an exposure value range of 0 to 1. Standard deviation classes in each map are followed by the percent of the state occurring in that class.

The potential for new wind development is highest in eastern and southeastern Wyoming (Figure 5), and obviously exposure of migration concentration areas is also greatest within these areas of the state (Figure 6). The spatial patterns in exposure varied among the four bird groups. For example, the highest exposures for grassland birds were mainly clustered in southeast Wyoming (Figure 6g), while high exposures for raptors were well-distributed along ridges throughout areas with high wind development potential (Figure 6e). Uncertainty in exposure to wind development ranged up to a standard deviation of 0.35 for wetland and riparian birds and 0.45 for raptors and sparse grassland birds (Figure 6), on an exposure scale of 0 to 1 (Figure 6).

Wind development potential was categorized as high or very high across 14.7% of Wyoming (Figure 5). Important migratory concentration areas for each of the four bird groups were exposed to only portions of this area of high development potential (Figure 6; Table 8). Sparse grassland bird important migration areas had the highest percent overlap with high wind potential areas and riparian birds the lowest (Table 8). Seventy-three percent of the high wind potential area overlaps with important migration areas, when considered across all bird groups (Table 8). This overlap is less for each individual group, and the individual values do not sum to the total because there is spatial overlap among migration areas for the various groups (Table 8). The 73% of the wind potential area that overlaps with important migration areas corresponds with 13.2% of the important migration areas, across all four groups (Table 8). Uncertainty in the two calculated percentages, represented by 95% confidence intervals, was low for all groups except sparse grassland birds (Table 8).

**Table 8:** Spatial overlap between the highest migration concentration areas and highest wind development potential areas, as represented by the models’ top two quantiles, including A) percent of the highest concentration migration areas that overlap with the highest wind potential areas and B) percent of the highest potential wind areas that overlap with the highest migration concentration areas.

Bird group	A) Percent of migration area		B) Percent of wind area		
	Full model	All models Mean (95% CI)	Full model		All models Mean (95% CI)
Wetland birds	9.7	8.9 (7.9–9.9)	26.6	24.4 (21.6–27.2)	
Riparian birds	5.2	5.1 (4.1–6.1)	6.3	6.1 (4.9–7.3)	
Raptors	7.3	7.7 (6.7–8.7)	19.2	20.5 (17.9–23.1)	
Sparse grassland birds	18.8	10.9 (5.6–16.2)	52.6	30.4 (15.5–45.3)	
All groups combined	13.2	na	73.0	na	

Percentages are presented for the full model and as the mean and 95% confidence interval across the full and partial sensitivity analysis models.

## Discussion

Models of migratory concentration for wetland, riparian, and sparse grassland birds were consistently rated as accurate representations based on validation from experts and existing datasets. These models provide a much needed initial assessment to highlight important resources for migrants where little is currently known, as is the case in Wyoming. The model set can be updated as new information on migration patterns or improved spatial data layers become available, and can be expanded to predict concentrations for other groups of birds or for individual species. Our approach could be replicated elsewhere to fill critical data gaps for migratory birds.

Our migratory concentration models can be used to inform siting of wind developments and identify where mitigation may be needed. These models are well-suited for the preliminary site evaluations recommended by the U.S. Fish and Wildlife Service (USFWS) to identify possible conflicts with habitats or species of concern at a landscape scale [13]. The USFWS implements the Migratory Bird Treaty Act, which prohibits the killing or harming of migratory birds, and wind energy developers are required to comply with this statute on both public and private lands. Our models could also be used by federal land management agencies, such as the Bureau of Land Management, to support regional planning for wind development on public lands. Preliminary site evaluations can identify where development might be avoided, and our models could also inform other stages of the mitigation hierarchy, including identifying potential mitigation offset opportunities [91] – where some important migration areas might be protected from development following impacts to other important migration areas. In locations where migratory birds concentrate and wind development cannot be avoided, a number of onsite mitigation techniques can be used to minimize risk to birds [8], [13], [15]. First, pre-construction surveys characterize bird use of project areas and aid in turbine placement that minimizes contact with birds and other wildlife. Second, minimal use of red or white flashing lights on wind turbines and associated infrastructure is much less likely to ‘draw’ migrating birds into the rotor-swept zone. Also, power transmission lines pose collision threats to migrating birds and should be minimized and buried when possible. Finally, post-construction surveys can help identify high mortality areas where additional mitigation measures may be needed, such as turning off high-risk turbines during peak migration times [15]. Our landscape-scale models are not a substitute for pre- or post-construction studies within project areas; mortality rates may be site-specific and depend upon the siting of individual turbines [92].

The amount and location of exposure of migratory birds to wind development differed among the four groups of migrants. Not surprisingly, we noted the greatest potential exposure for sparse grassland birds, which use grasslands in the southeastern portion of Wyoming that havesome of the best wind resources. There was also the greatest uncertainty in exposure for sparse grassland birds, as that model relied heavily on grassland cover types, that when removed, shifted the important migration areas outside the geographic extent where wind development is anticipated. For sparse grassland birds, we expect that the overlap estimates with wind potential that are based on the full model including all three variables is the most accurate, due to the limited number of model factors. The raptor model performed poorly in validation against existing occurrence data, but we retained the model in the wind exposure analysis because it was rated well by experts and currently provides the only available spatially-synthesized information for raptor migration in Wyoming. The amount of exposure for raptors showed little variability among the full and partial models, suggesting that the model may offer a reasonable best estimate of exposure to wind development. Potential conflict was most limited for riparian birds, which are the most concentrated of the migrants, clustered primarily along valleys that generally have lower wind development potential. As a percentage of the total area of important migration concentration areas, overlap with the highest wind development potential areas was relatively low, ranging from 5.2 to 18.8%. However, impacts to these small relative percentages of the migration concentration areas could have a proportionally larger population impact. We expect that 60% or more of migrating individuals from each of these functional bird groups may be using the areas that we identified as most important (i.e., the top two model quantiles).

Our findings demonstrate that there are locations where wind facilities could be sited that may limit exposure of migratory birds to these developments. In 27.5% of the area classified as having high or very high potential for wind development, there was only low or moderate potential exposure of migratory concentration areas. Similarly, other studies have demonstrated that U.S. wind energy needs could be met by siting wind development in previously disturbed areas [93] and that mitigation requirements and associated costs could be greatly reduced by avoiding wind development in the most sensitive wildlife habitats [94]. Wyoming could exceed the U.S. Department of Energy’s wind energy goal for the state by 2662% even if development avoided sensitive biological areas [95], not including the migratory concentration areas presented here.

Our models of migration concentration are limited by the availability and quality of bird occurrence data, predictive GIS layers, and information on migration behavior. The models represent where migration concentration is expected to be highest in most years based on fixed factors, but migration varies among years and is influenced by weather and variation in food resources. The Wyoming bird species occurrence databases contained thousands of records, but only a small portion of these corresponded to the migratory season. Further, most were opportunistic observations rather than data obtained through systematic, unbiased sampling, and some areas of Wyoming were underrepresented. The models were evaluated by experts and assessed against occurrence data, and a logical next step toward improving the models would be structured field validation. Although limited by available data, our migratory concentration models provide a useful spatial synthesis of the information that currently exists and fill a critical gap for landscape-scale planning.

We used the best available knowledge concerning factors that affect migration patterns to create the migration concentration models. For most bird groups, our modeling approach appears to have been effective, based on validation results, but the raptor model is a possible exception.

The raptor model performed well in the expert validation but poorly when compared to existing occurrence data. There may be factors influencing fall migration movement patterns that are not currently understood well enough, or the datasets or methods we used to represent important factors may be limited in some way. An alternative explanation is that the occurrence data are better suited for models of stopovers than for movement, as most observers record birds when they are perching or foraging. For all of the models, we selected model factors, modifiers, and weights based on literature review and expert knowledge. The sensitivity analysis showed that some of these model terms had a greater influence on the outcomes than others. The models were generally most sensitive to factors that affected a relatively large geographic extent (e.g., buffer in the wetland bird model or updrafts and thermals in the raptor model), or because they had been identified as the factor of greatest importance and been valued accordingly. Obviously we would expect the models to be influenced by key factors in this way, yet the sensitivity analysis remains informative because it provides an estimate of the degree to which model results may change given changes in knowledge or assumptions and it provides a range of uncertainty that can be compared with our estimates of bird migration concentration patterns. For the wetland and riparian bird models that had the most model terms, there was very little variation in model results when some modifiers were dropped from the model (e.g., cottonwood or willow abundance for the riparian bird model). This suggests that this modeling approach is robust to minor modifications and that it may be most important to focus on the key factors believed to drive migratory patterns. The sparse grassland bird model was the most sensitive to removal of model terms likely because of the small number of factors, and because we know the least about this group’s migration patterns and behavior.

Wind development has the potential to impact bird populations far beyond the localities of wind facilities if development occurs without regard for migrating birds that may breed or overwinter in other parts of the world. Although migratory birds are protected under international treaties, limited datasets are currently available representing migration at a scale useful to guide development or target protection. Our migratory concentration models provide preliminary spatial data to companies, land management agencies, and others planning for wind development at a useful landscape scale across the state of Wyoming. The migratory concentration models can also help to target conservation efforts for migratory birds, such as conservation easements and stopover habitat enhancements, and our methods could be replicated in other locations or for other groups or species of birds.
